# PRKAR1A and SDCBP Serve as Potential Predictors of Heart Failure Following Acute Myocardial Infarction

**DOI:** 10.3389/fimmu.2022.878876

**Published:** 2022-05-03

**Authors:** Qixin Chen, Lina Su, Chuanfen Liu, Fu Gao, Hong Chen, Qijin Yin, Sufang Li

**Affiliations:** ^1^ Department of Cardiology, Beijing Key Laboratory of Early Prediction and Intervention of Acute Myocardial Infarction, Center for Cardiovascular Translational Research, Peking University People’s Hospital, Beijing, China; ^2^ Department of Cardiac Surgery, Yale School of Medicine, Yale University, New Haven, CT, United States; ^3^ Ministry of Education Key Laboratory of Bioinformatics, Research Department of Bioinformatics at the Beijing National Research Center for Information Science and Technology, Center for Synthetic and Systems Biology, Department of Automation, Tsinghua University, Beijing, China

**Keywords:** leukocytes, acute myocardial infarction, biomarkers, cell–cell communication, heart failure

## Abstract

**Background and Objectives:**

Early diagnosis of patients with acute myocardial infarction (AMI) who are at a high risk of heart failure (HF) progression remains controversial. This study aimed at identifying new predictive biomarkers of post-AMI HF and at revealing the pathogenesis of HF involving these marker genes.

**Methods and Results:**

A transcriptomic dataset of whole blood cells from AMI patients with HF progression (post-AMI HF, n = 16) and without progression (post-AMI non-HF, n = 16) was analyzed using the weighted gene co-expression network analysis (WGCNA). The results indicated that one module consisting of 720 hub genes was significantly correlated with post-AMI HF. The hub genes were validated in another transcriptomic dataset of peripheral blood mononuclear cells (post-AMI HF, n = 9; post-AMI non-HF, n = 8). PRKAR1A, SDCBP, SPRED2, and VAMP3 were upregulated in the two datasets. Based on a single-cell RNA sequencing dataset of leukocytes from heart tissues of normal and infarcted mice, PRKAR1A was further verified to be upregulated in monocytes/macrophages on day 2, while SDCBP was highly expressed in neutrophils on day 2 and in monocytes/macrophages on day 3 after AMI. Cell–cell communication analysis *via* the “CellChat” package showed that, based on the interaction of ligand–receptor (L–R) pairs, there were increased autocrine/paracrine cross-talk networks of monocytes/macrophages and neutrophils in the acute stage of MI. Functional enrichment analysis of the abovementioned L–R genes together with PRKAR1A and SDCBP performed through the Metascape platform suggested that PRKAR1A and SDCBP were mainly involved in inflammation, apoptosis, and angiogenesis. The receiver operating characteristic (ROC) curve analysis demonstrated that PRKAR1A and SDCBP, as well as their combination, had a promising prognostic value in the identification of AMI patients who were at a high risk of HF progression.

**Conclusion:**

This study identified that PRKAR1A and SDCBP may serve as novel biomarkers for the early diagnosis of post-AMI HF and also revealed their potentially regulatory mechanism during HF progression.

## Introduction

Heart failure (HF) has become a public health concern worldwide. Although the survival rate of patients with HF has improved due to the recent developments in therapeutic strategies, the 5-year mortality of HF remains within 50%, indicating the necessity of further attention to the prevention and treatment of HF (1, 2). Acute myocardial infarction (AMI), as a serious type of coronary heart disease, is a major contributor to the HF progression (3). A growing body of evidence showed that approximately 25% of survivors after ST-segment-elevation myocardial infarction developed HF (4–7). Therefore, early diagnosis of AMI patients who are at a high risk of HF progression is vital to reducing HF incidence.

Biomarkers, characterized by low expenditure, low risk, and rapid detection, may provide essential information for the complicated pathogenesis of post-AMI HF and are therefore extremely advantageous for the early identification of HF, as well as a better prognosis of AMI (8). Although several genes, such as natriuretic peptides (NPs), galectin-3, and soluble suppression of tumorigenicity-2 (sST2), were reported as biomarkers of HF, their reliability remains controversial (9, 10). Therefore, it is essential to explore further reliable biomarkers for the early prediction of post-AMI HF.

Genome-wide transcriptome analysis (e.g., microarray) provides an unbiased and comprehensive view of gene expression characteristics in specific disease models, which is highly efficient in the screening of biomarkers. Differential gene expression analysis is mainly used to identify key genes, while it may cause the loss of essentially biological information. A systems biology method termed weighted gene co-expression network analysis (WGCNA) is thus introduced into the analysis of high-throughput data to investigate key genes (termed hub genes) related to diseases or clinical traits (11–16). WGCNA can calculate the correlation between genes and sample traits, recognize clinically associated gene modules, and identify hub genes for further validation (17). Single-cell RNA sequencing (scRNA-seq) allows the investigation of gene expression and the identification of transcriptionally different subpopulations at a single-cell resolution (18). Additionally, scRNA-seq can provide important information of the cell–cell communication between cell subpopulations mediated through ligand–receptor (L–R) interactions (19).

In the present study, we aimed to identify predictive biomarkers of HF following AMI using WGCNA and to explore the mechanisms of post-AMI HF involving the candidate marker genes at the single-cell resolution. Based on the circulating transcriptome datasets of GSE11947 (for WGCNA) and GSE59867 (for validation), upregulated protein kinase cAMP-dependent type I regulatory subunit alpha (PRKAR1A), syndecan-binding protein (SDCBP), EVH1-domain-containing protein 2 (SPRED2), and vesicle-associated membrane protein-3 (VAMP3) were proved to be highly correlated with HF progression after AMI. Based on the scRNA-seq of the GSE135310 dataset, complicated autocrine/paracrine cross-talk networks were found between monocytes/macrophages and neutrophils. These two cell subpopulations might be related to the upregulation of circulating PRKAR1A and SDCBP in the acute phase of AMI. Monocyte/macrophage- or neutrophil-related L–R genes together with PRKAR1A or SDCBP may be primarily associated with inflammatory response, apoptosis, and angiogenesis. Concordantly, upregulated PRKAR1A and SDCBP in the abovementioned three datasets were finally confirmed to be predictive biomarkers for AMI patients who were at a high risk of HF progression.

## Materials and Methods

### Data Processing

**Figure 1:**
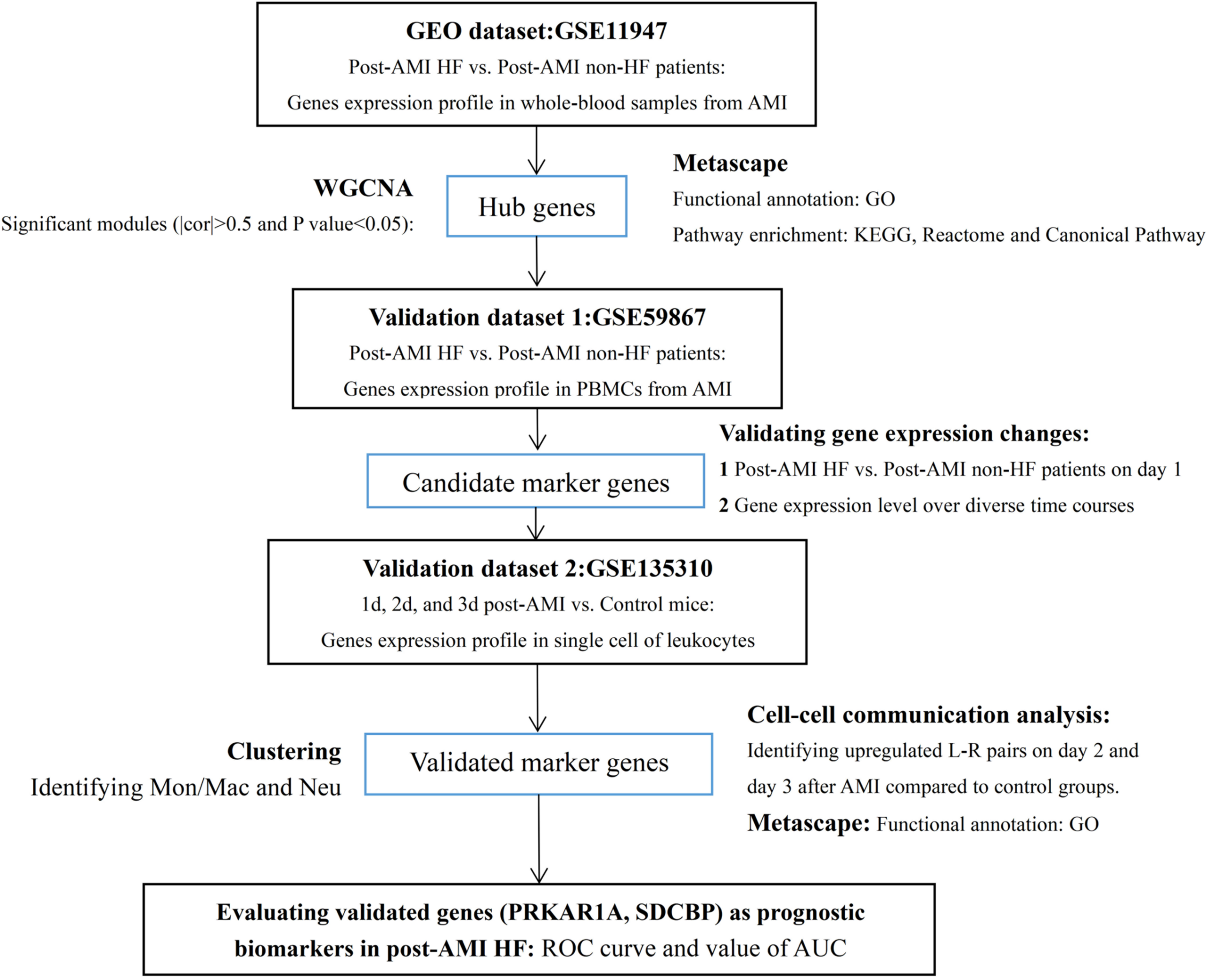
Study flowchart. GEO, Gene Expression Omnibus; AMI, acute myocardial infarction; HF, heart failure; PBMCs, peripheral blood mononuclear cells; WGCNA, weighted gene co-expression network analysis; cor, correlation coefficient; GO, Gene Ontology; KEGG, Kyoto Encyclopedia of Genes and Genomes; Mon/Mac, monocytes/macrophages; Neu, neutrophils; L–R pairs, ligand–receptor pairs; ROC, receiver operating characteristic; AUC, area under the ROC curve.

The study flowchart is presented in **Figure 1**. In the present study, two microarray datasets (GSE11947 and GSE59867) and a scRNA-seq dataset (GSE135310) were selected from the Gene Expression Omnibus (GEO) database (https://www.ncbi.nlm.nih.gov/geo). In the GSE11947 dataset, the gene expression profiles of whole blood cells from 32 patients within 12 h after AMI with developed HF [mean ejection fraction (EF) = 35%; n = 16] or without HF (mean EF = 63%; n = 16) during the 1-month follow-up were acquired using the oligonucleotide microarrays, representing 25,000 genes. These two age- and sex-matched groups were not different in terms of reperfusion time, final coronary flow, multivessel disease, history of infarction, cardiovascular risk factors, and treatment. This study was approved by the local ethics committee (Comité National d’Ethique de la Recherché, CNER), and written informed consent was obtained from all patients (20). The locally weighted scatterplot smoothing (LOWESS) of data was performed using the Acuity software (Molecular Devices, San Jose, CA, USA) (21). Genes detected in at least 50% of samples were included, and a total of 13,692 genes were finally involved in the subsequent analysis (20). In the GSE59867 dataset, gene expression data of peripheral blood mononuclear cells (PBMCs) from patients within 1 day (1 d), 4–6 days (4–6 d), 30 days (30 d), and 180 days (180 d) after AMI with developed HF (mean EF = 39.3%, 1 d: n = 9, 4–6 d: n = 9, 30 d: n = 8, 180 d: n = 9) or without HF (mean EF = 66.8%, 1 d: n = 8, 4–6 d: n = 6, 30 d: n = 8, 180 d: n = 8) during a 6-month follow-up were processed for background correction, log_2_ transformation, and quantile normalization using the Robust Multi-array Average algorithm. The demographic characteristics of post-AMI HF (n = 9) and non-HF (n = 8) patients from the GSE59867 dataset are listed in **Supplementary Table 1**. This study was approved by the local Ethics Committees of the Medical University of Warsaw and Medical University of Bialystok and was conducted in accordance with the principles of the Declaration of Helsinki. All participants gave written informed consent (22). For the GSE135310 dataset, single-cell gene expression data of CD45+ leukocytes from heart tissues of normal (control) (n = 5) and infarcted mice within 1 d (n = 5), 2 d (n = 5), and 3 d (n = 5) after AMI were processed using Cell Ranger™ 3.0.1 pipelines (http://10xgenomics.com). All animal studies used conform to the Directive 2010/63/EU of the European Parliament and have been approved by the appropriate local authorities (Regierung von Unterfranken, Würzburg, Germany, Akt.-Z. 55.2-DMS-2532-2-743 2 and Akt.-Z. 55.2-DMS-2532-2-865) (23).

### Co-Expression Network Construction

A co-expression network of the gene expression dataset GSE11947 was constructed using the “WGCNA” package in R software. After screening using the WGCNA algorithm and sorting by median absolute deviation (MAD), 8,349 genes were retained for pickSoftThreshold function. The scale-free topology fit index of 0.8 was used to find out an appropriate soft threshold power. Filtered gene expression data were processed using the power adjacent function of Pearson’s correlation matrix to convert data into a topological overlap matrix (TOM), and the corresponding dissimilarity (1-TOM) was calculated. Genes with similar expression profiles were merged into the same modules using the DynamicTreeCut algorithm (cutHeight = 0.3, deepSpilt = 0.2, and minModularSize = 20).

### Identification of Clinically Significant Modules

Module eigengenes (MEs) were defined as the first principal component of each gene module, whose expression was regarded as the representative of gene expression profiles in a module. Gene significance (GS) indicated the correlation between gene expression and clinical trait. Module significance (MS) represented the average GS for all the genes classified into a module, indicating the correlation between the selected module and the clinical trait. The module with absolute correlation coefficient (|cor|) > 0.5 and, after Bonferroni correction, Q-value < 0.05, was considered to be significantly associated with the disease trait.

### Identification of Hub Genes

Module membership (MM) was defined as the correlation between the ME and the gene expression profile in a selected module. GS representing the correlation between a gene and the disease trait in combination with MM was used to identify hub genes. Genes with |GS| >0.2 and |MM| > 0.8 were defined as the most highly connected intramodular hub genes (24).

### scRNA-Seq Analysis

Single-cell samples of control and AMI mice were merged for the following analyses. Standard processing steps, including filtering, identification of highly variable genes, dimensionality reduction, and clustering of the cells, were performed using the “Seurat” package in the R software (ver. 4.0.1). The low-quality cells expressing <200 genes and genes expressed in <3 cells were excluded. To remove the possible doublets, cells expressing >2,500 genes were excluded. Cells with >5% mitochondrial genes were regarded as poor-quality and were removed. Finally, 13,593 genes among 2,224 leukocytes were screened from 3,035 cells and selected for further analyses. To normalize the data, the gene expression levels of the remaining cells were multiplied by 10,000, and the results were log-transformed. Highly variable genes were selected using standard variation and were used in the downstream analyses. To scale the data, linear transformation, a standard preprocessing step prior to dimensionality reduction, was applied to the data in order to shift the mean expression to 0 and variance to 1. Principal component analysis (PCA) was conducted based on the top 2,000 highly variable genes for dimensionality reduction, and 30 significant principal components were chosen. Clustering in PCA was performed using the graph-based clustering approach with a resolution of 0.1. The Louvain algorithm was used to group cells into different clusters. t-Distributed stochastic neighbor embedding (tSNE) was applied for the two-dimensional visualization of the clustering cells. Differentially expressed genes (DEGs) in each cluster were identified by the Wilcoxon test. Genes with log_2_^FC^ (fold change, FC) > 0.25 and adjusted P-value (pvals_adj) < 0.05 were considered as significant DEGs.

### Cell–Cell Communication Analysis

The “CellChat” package in the R software (ver. 1.1.3) was applied to infer and visualize the cell–cell communication between different clusters based on the L–R interaction (25). Briefly, normalized scRNA-seq data by the “Seurat” package were imported into the “CellChat” package. Considering specific cell types as receiver, in order to identify the upregulated L–R pairs, we compared the interactions of the control group and AMI group.

### Functional Enrichment Analysis

Metascape (https://metascape.org/) is a web-based portal designed to provide a comprehensive gene list annotation and analysis resource for experimental biologists. The Gene Ontology (GO) of biological process (BP) enrichment analysis and pathway analyses, including Kyoto Encyclopedia of Genes and Genomes (KEGG), Reactome gene set, and canonical pathway, were performed on genes of interest through Metascape. Enriched GO-based bp terms and pathways were considered statistically significant when the P-value was <0.05.

### Statistical Analysis

For making multiple comparisons to identify clinically significant modules, Bonferroni correction was conducted using the “qvalue” package in the R software (ver. 2.22.0). The Q-value was calculated based on the P-value. A Q-value <0.05 was considered statistically significant. The expression levels of hub genes were presented as mean ± standard error of the mean (SEM). The Mann–Whitney U test was utilized to compare the discrepancy in gene expression levels between two groups. The receiver operating characteristic (ROC) curve analysis was carried out based on gene expression levels. The area under the ROC curve (AUC) was calculated to assess the specificity and sensitivity of single genes and their combination *via* the binary logistic regression analysis. GraphPad Prism (ver. 8.0) and MedCalc (ver. 19.1) software were used for the statistical analysis. A P-value < 0.05 was considered statistically significant.

## Results

### Co-Expression Network Construction and Identification of Significant Modules

The co-expression network was constructed by WGCNA. In the GSE11947 dataset, the gene expression profiles of whole blood cells from 32 patients within 12 h after AMI with HF progression (n = 16) or without progression (n = 16) during the 1-month follow-up were obtained. A total of 8,394 genes and 32 samples were clustered using the average linkage method and the Pearson’s correlation method. The final power of 22 was chosen as the soft-thresholding parameter to ensure that the network was scale free (**Supplementary Figure 1A**). A total of 16 modules were identified, and the gray module containing genes that could not be clustered into other modules was discarded (**Supplementary Figure 1B**).

**Figure 2:**
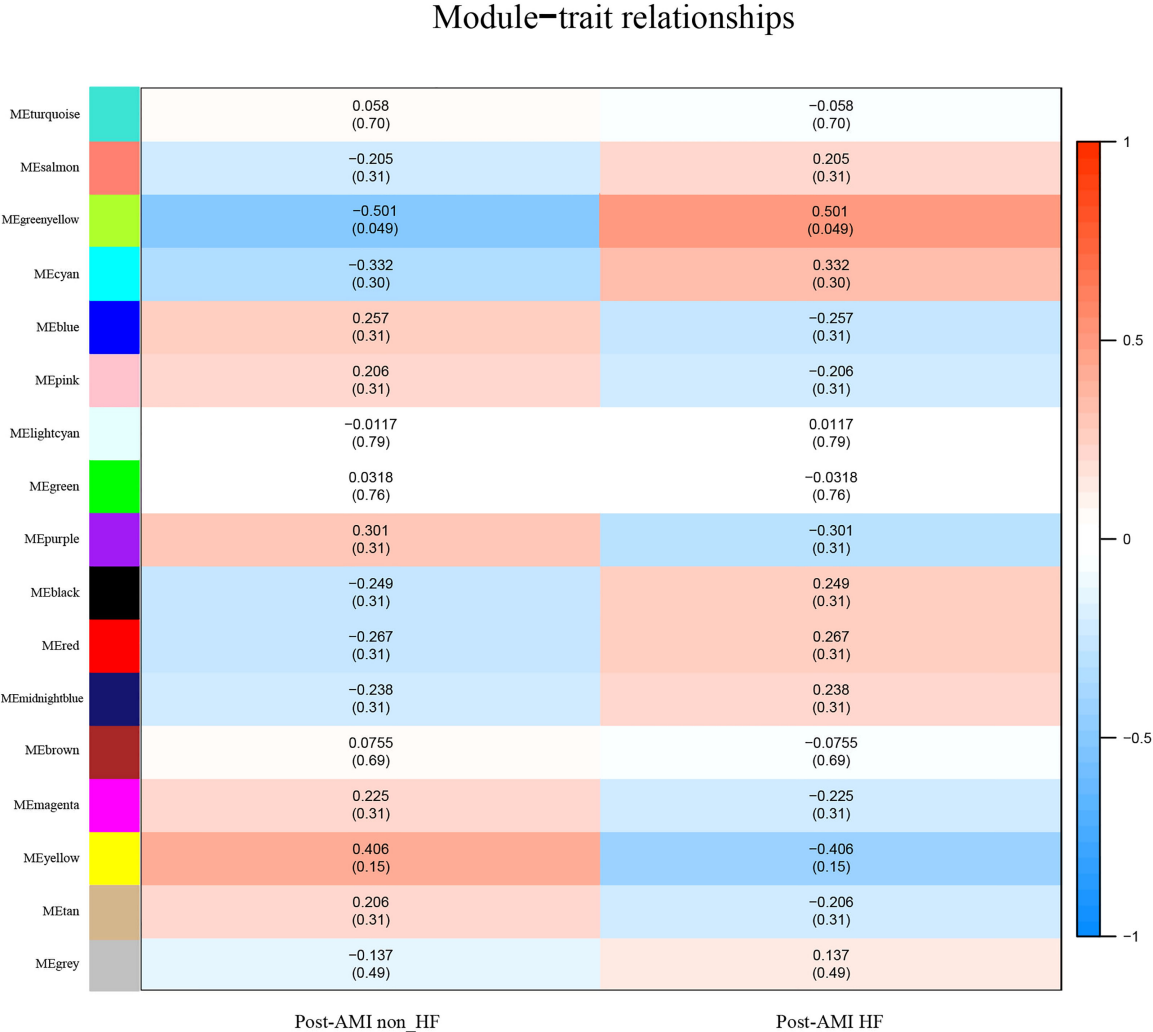
Identification of highly correlated modules of post-AMI HF. Heatmap of the association between module eigengenes and HF progression after AMI. The correlation coefficient (cor) and Q-value are presented in each cell. The greenyellow module was found to be significantly correlated with post-AMI HF according to |cor|>0.5 and Q-value < 0.05. Red indicates a positive correlation between two modules, and blue indicates a negative correlation. AMI, acute myocardial infarction; HF, heart failure.

The greenyellow module showed a positive correlation with post-AMI HF according to the Gleason score (**Figure 2**), which would be identified as the clinically significant module for the following analyses. Additionally, the co-expression similarity of the 16 modules was quantified by measuring the correlations between MEs (**Supplementary Figure 2**).

### GO and Pathway Enrichment Analyses

Genes of the clinically significant module were selected to perform GO and pathway enrichment analyses. The 218 genes corresponding to the greenyellow module (**Supplementary Table 2**) were imported into the Metascape platform (https://metascape.org/). Genes of the greenyellow module were implicated in the BPs of myeloid leukocyte activation, response to growth factors, negative regulation of cellular catabolic process, and response to endoplasmic reticulum stress (**Supplementary Figure 3A**, **Supplementary Table 3**), as well as pathways, such as NOTCH4 pathway, cellular responses to stress pathway, IL8 CXCR1 pathway, syndecan-2 pathway, integrin A4B1 pathway, P38 alpha/beta downstream pathway, P53 downstream pathway, and sphingolipid signaling pathway (**Supplementary Figure 3B**, **Supplementary Table 4**). The abovementioned GO-enriched terms and pathways suggested that genes in significant modules had a close relationship with the pathogenesis of AMI.

### Identification of Hub Genes

Based on the cutoff criteria of |MM| >0.8 and |GS| >0.2, 68 out of 218 genes of the greenyellow module were identified as the most highly connected intramodular hub genes (**Supplementary Table 5**). The MM and GS in the greenyellow module are shown in **Supplementary Figure 4**.

### Validation of Key Bio-Correlated Genes

**Figure 3:**
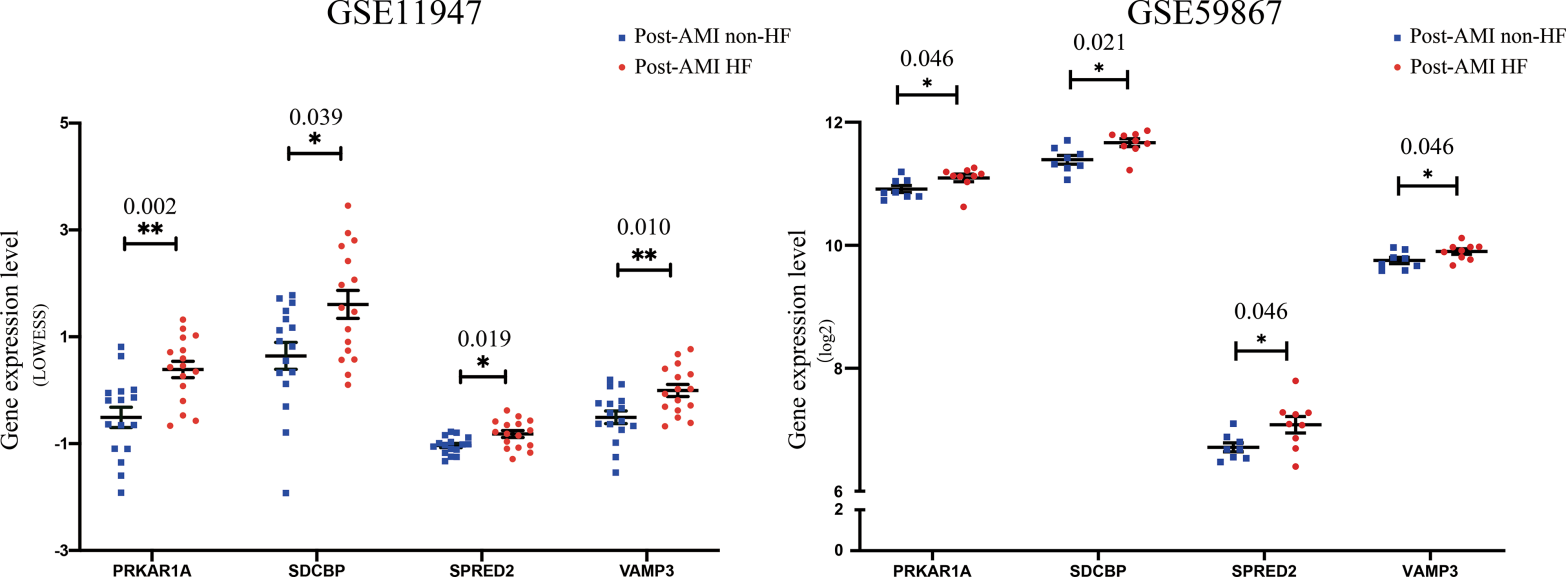
Validation of the expression levels of key bio-correlated genes in post-AMI HF and post-AMI non-HF patients based on GSE11947 and GSE59867 datasets. GSE11947: HF n = 16, non-HF n = 16; GSE59867: HF n = 9, non-HF n = 8. P-value was shown above the asterisk (*). *P < 0.05, **P < 0.01. Data are presented as mean ± SEM. AMI, acute myocardial infarction; HF, heart failure; SEM, standard error of mean.

To further determine the key bio-correlated genes, the overlapped hub genes in the GSE11947 and GSE59867 datasets (AMI for 1 day) and their expression levels were identified and tested. The results showed that there were 145 overlapped genes in the two datasets (**Supplementary Table 6**). Notedly, 4 genes, namely, PRKAR1A, SDCBP, SPRED2, and VAMP3, were consistently upregulated in patients with and without HF progression in both datasets (**Figure 3**), while other genes that showed no significant expression change or an opposite trend of expression change in the two datasets were excluded.

**Figure 4:**
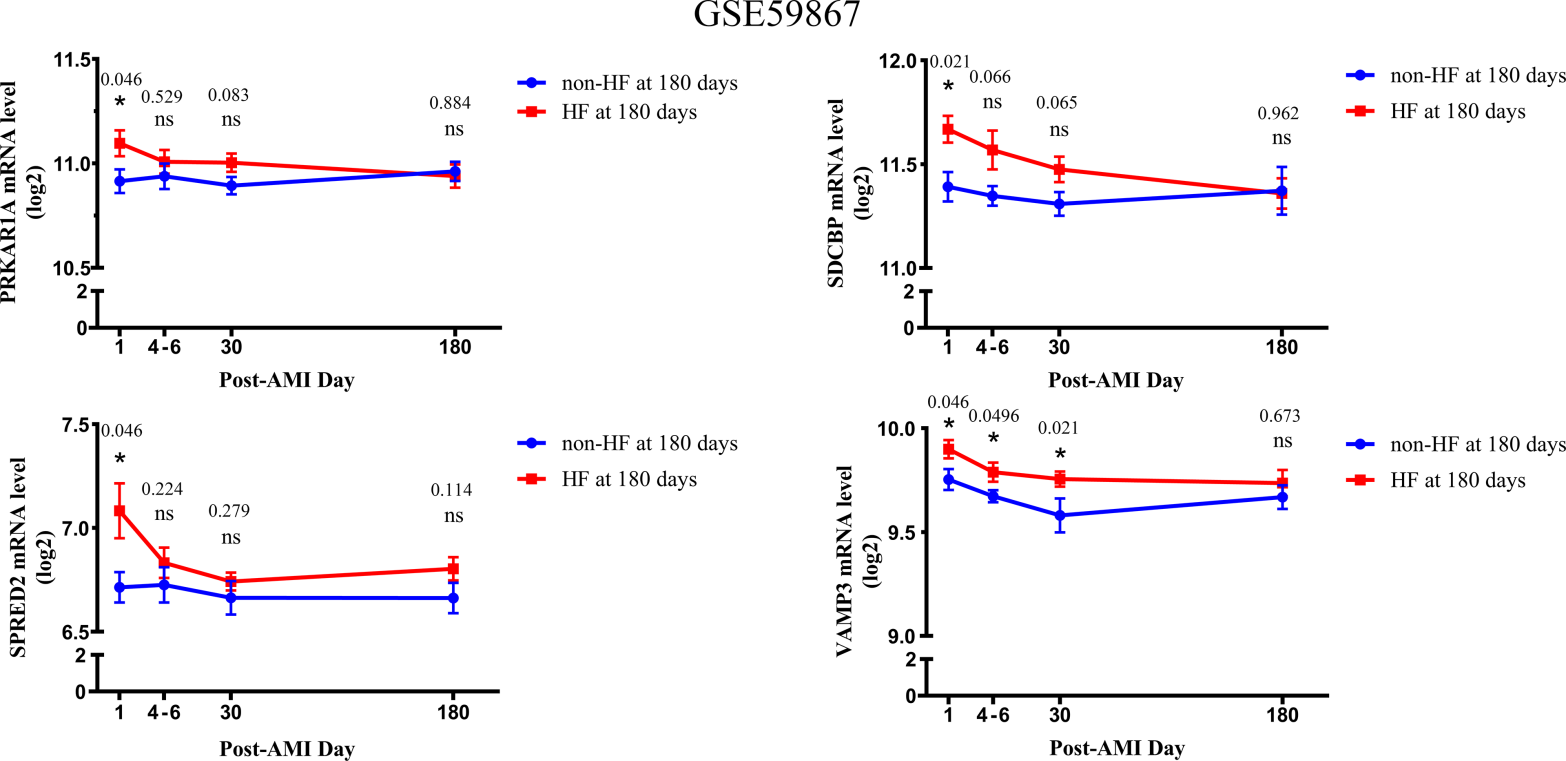
The changes in expression levels of key bio-correlated genes over time, following AMI in the GSE59867 dataset. The changes in gene expression levels were observed in the HF and non-HF groups at different time points after AMI (1d: n = 9 vs. 8, 4–6 days: n = 9 vs. 6, 30 days: n = 8 vs. 8, 180 days: n = 9 vs. 8). The P-value was shown above the asterisk (*). *P < 0.05; n.s., no significance vs. non-HF group. Data are presented as mean ± SEM. AMI, acute myocardial infarction; HF, heart failure; SEM, standard error of mean.

Based on the GSE59867 dataset, the dynamic changes of 4 validated genes at 1 d, 4–6 d, 30 d, and 180 d after AMI in patients with and without HF progression were further investigated. The results indicated that the expression levels of PRKAR1A, SDCBP, SPRED2, and VAMP3 exhibited the most significant increase at 1 d after AMI, which then gradually decreased in HF patients (**Figure 4**). Moreover, the VAMP3 expression level in HF patients was higher than that in non-HF patients at 1 d, 4–6 d, and 30 d after AMI. Meanwhile, the expression levels of 4 genes showed no significant difference between HF and non-HF patients at 180 d after AMI. Taken together, these results suggested the early predictive potential of PRKAR1A, SDCBP, SPRED2, and VAMP3 in HF progression after AMI.

### Discrimination of Cell Source of Validated Genes at Single-Cell Resolution

Leukocytes accounted for less than 1% of whole blood cells, and they could infiltrate the injured myocardium within a few hours or days after the onset of AMI, triggering inflammatory/immune response and subsequently cardiac remodeling (26). Types of leukocytes are monocytes, granulocytes (neutrophils, eosinophils, and basophils), and lymphocytes (T cells and B cells). To discriminate the specific cell type related to the upregulation of PRKAR1A, SDCBP, SPRED2, and VAMP3 at the early stage of AMI, scRNA-seq data of CD45+ leukocytes from infarcted and normal mice (GSE135310) were extracted to perform cell clustering, cell type definition, and differential gene expression analysis.

**Figure 5:**
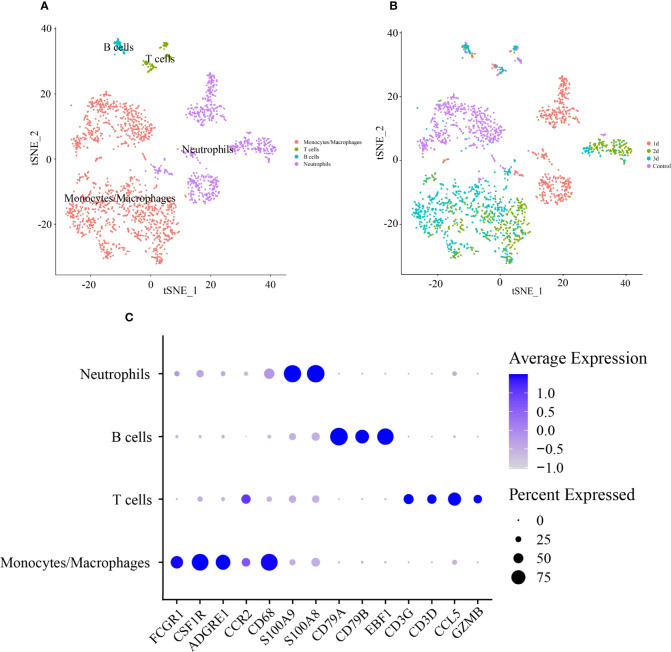
Identification of cell type of CD45+ lymphocytes in infarcted cardiac tissues of mice. **(A)** tSNE visualization of cell type in heart tissues of control and infarcted mice. **(B)** tSNE visualization of lymphocytes in heart tissues of control mice at 1 day (1 d), 2 days (2 d) and 3 days (3 d) after AMI. **(C)** Dot plot of canonical markers of different cell types. Dot size reflects the percentage of cells expressing the markers in each cell type. The scale color represents the gene expression levels from low to high.

Based on a PCA of 30 and resolution of 0.1, 2,224 CD45+ leukocytes were classified into 8 clusters (**Supplementary Figure 5A**). The cell types were defined according to the expression levels of canonical cell type markers in each cluster. Cells in clusters 0 and 1 were enriched with markers of monocytes/macrophages (Cd68, Adgre1, Fcgr1, Csfr1, and Ccr2). Clusters 2, 3, and 4 included highly expressed markers of neutrophils (S100a8 and S100a9). Markers of T cells (Cd3d, Ccl5, Cd3g, and Gzmb) and B cells (Cd79a, Cd79b, and Ebf1) were highly expressed in clusters 5 and 6, respectively (**Figure 5C**). The expression levels of representative marker genes in each cluster are also shown in **Supplementary Figure 5B**. Then, cell type identity was assigned to each cluster (**Figure 5A**). The distribution of all CD45+ leukocytes in control and infarcted mice at 1 d, 2 d, and 3 d is illustrated in **Figure 5B**, indicating an obviously stage-based distribution of monocytes/macrophages and neutrophils.

**Figure 6:**
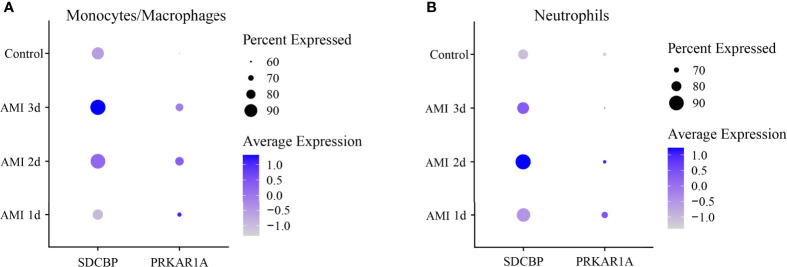
Dot plot of expression profiles of PRKAR1A (**A**) and SDCBP (**B**) in monocytes/macrophages and neutrophils at different time points after AMI. Dot size reflects the percentage of cells expressing the markers in each cell type. The scale color represents the gene expression levels from low to high. AMI, acute myocardial infarction.

Then, DEGs in a subset of monocytes/macrophages and a subset of neutrophils between control and 1 d, 2 d, and 3 d after AMI were analyzed. It was revealed that PRKAR1A was highly expressed in monocytes/macrophages at 2 d after MI, while SDCBP was enriched in neutrophils at 2 d after MI and in monocytes/macrophages at 3 d after MI (**Figure 6**, **Supplementary Table 7**). The expression levels of SPRED2 and VAMP3 in monocytes/macrophages or neutrophils were not significantly different between control and 1 d, 2 d, or 3 d after AMI. Thus, monocytes/macrophages and neutrophils could be the major contributors to the upregulation of PRKAR1A and SDCBP in the acute stage of MI, rather than being responsible for the changes in expression levels of SPRED2 and VAMP3. After that, we concentrated on the pathophysiological characteristics of monocytes/macrophages at 2 d and 3 d after AMI, as well as those of neutrophils at 2 d after AMI.

### Investigation of the Cell–Cell Communication Among Subtypes of Leukocytes

**Figure 7:**
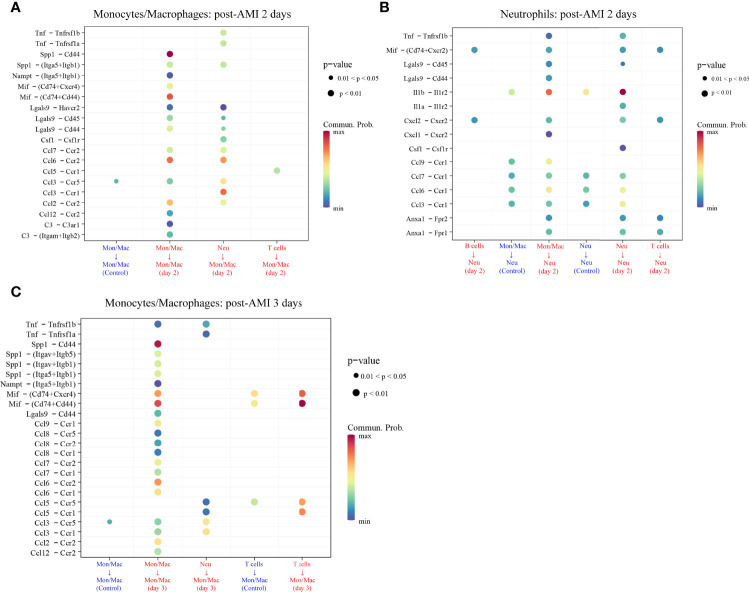
Upregulated ligand–receptor signaling received by monocytes/macrophages (Mon/Mac) on days 2 **(A)** and 3 **(C)** after AMI and neutrophils (Neu) on days 2 after AMI **(B)**. Cell–cell communication of recipient Mon/Mac and Neu was analyzed by the “CellChat” package. Dot size reflects the P-value of the signaling. The scale color represents the level of communication probability from minimum to maximum. AMI, acute myocardial infarction; Neu, neutrophils.

The cell–cell communication can be reflected by the expression levels of ligands and their corresponding receptors. Based on scRNA-seq data of the GSE135310 dataset, the cell–cell communication analysis of recipient monocytes/macrophages and neutrophils was performed *via* the “CellChat” package. As displayed in **Figure 7** and **Supplementary Table 8**, at 2 d and 3 d after AMI, monocytes/macrophages received 20 and 24 upregulated L–R signaling pathways sent by monocytes/macrophages, neutrophils, and T cells, respectively. Meanwhile, neutrophils received 15 upregulated signaling pathways sent by B cells, monocytes/macrophages, neutrophils, and T cells at 2 d post-AMI. Collectively, these results revealed the complex autocrine/paracrine networks of monocytes/macrophages and neutrophils in the early stage of MI.

### Mechanism Analysis of PRKAR1A and SDCBP Involved in Post-AMI HF

As displayed in **Figure 7**, there were 29 L–R pair genes in the upregulated signaling sent or received by monocytes/macrophages on day 2 after AMI, and 35 L–R pair genes on day 3 after AMI. Meanwhile, 40 L–R pair genes were found in the upregulated signaling sent or received by neutrophils on day 2 after AMI.

To reveal the potentially regulatory roles of PRKAR1A and SDCBP in the progression of HF after AMI, the abovementioned identified L–R pair genes, along with PRKAR1A or SDCBP, were imported into the Metascape platform to perform the analysis of GO BP. The results showed that on day 2 after AMI, PRKAR1A in monocytes/macrophages was mainly involved in the BP of leukocyte proliferation, cellular extravasation, positive regulation of immune effector process, and positive regulation of chemokine (C–X–C motif) ligand 2 production (**Supplementary Table 9**); SDCBP in neutrophils was closely associated with the regulation of leukocyte migration, regulation of mononuclear cell proliferation, positive regulation of leukocyte differentiation, positive regulation of chemokine production, and regulation of the myeloid cell apoptotic process (**Supplementary Table 10**). Besides, on day 3 after AMI, SDCBP in monocytes/macrophages was primarily associated with the regulation of leukocyte chemotaxis, positive regulation of cell adhesion, and angiogenesis (**Supplementary Table 11**).

### Evaluation of PRKAR1A and SDCBP as Prognostic Biomarkers

**Figure 8:**
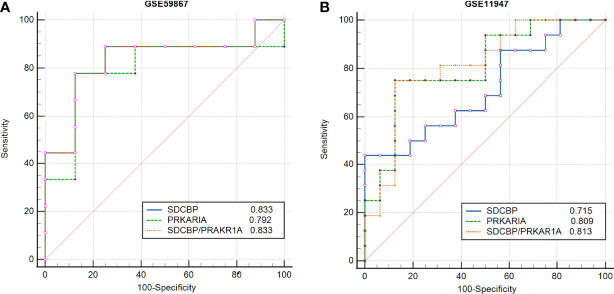
Receiver operating characteristic (ROC) curve analysis of potential biomarkers. ROC curves of PRKAR1A, SDCBP, and the combination of SDCBP/PRKAR1A, and the areas under the ROC curves were presented based on the expression levels of GSE59867 **(A)** and GSE11947 **(B)** datasets, respectively.

To evaluate the potential prognostic value of the four validated genes in **HF progression after AMI,** ROC curve analysis w**as performed on the** GSE11947 (n = 32) and GSE59867 (n = 17) datasets. The AUC values of PRKAR1A and SDCBP in the GSE11947 dataset were 0.809 and 0.715 and were 0.792 and 0.833 in the GSE59867 dataset, respectively (**Figures 8A, B** as well as **Tables 1** and **2**), indicating a promising prognostic value of these four genes. The AUC values of the combination of these two genes (SDCBP/PRKAR1A) are shown in **Figures 8A, B** as well as **Tables 1** and **2**, while the combination of SDCBP and PRKAR1A (GSE11947, AUC = 0.813; GSE59867, AUC = 0.833) manifested the best prognostic value in identifying AMI patients who were at a high risk of HF progression.

**Table 1:** Receiver operating characteristic curves in GSE11947.

	AUC	95%CI	P-value	Specificity (%)	Sensitivity (%)
SDCBP	0.715	0.528~0.860	0.0207	100.00	43.75
PRKAR1A	0.809	0.654~0.963	0.0001	87.50	75.00
SDCBP/PRKAR1A	0.813	0.658~0.967	0.0001	87.50	75.00

AUC, area under the curve; CI, confidence interval.

**Table 2:** Receiver operating characteristic curves in GSE59867.

	AUC	95%CI	P-value	Specificity (%)	Sensitivity (%)
SDCBP	0.833	0.577~0.966	0.0026	87.50	77.78
PRKAR1A	0.792	0.530~0.946	0.0207	87.50	77.78
SDCBP/PRKAR1A	0.833	0.577~0.966	0.0026	87.50	77.78

AUC, area under the curve; CI, confidence interval.

## Discussion

High-throughput transcriptomic technology is a potential strategy to identify biomarkers for AMI patients who were at a high risk of HF progression and to explore genes involved in HF. In the present study, we analyzed the gene expression profiles of whole blood cells from patients within 12 h after the onset of AMI in the GSE11947 dataset using the WGCNA algorithm. The identified hub genes correlated with developed HF at follow-up were validated in the GSE59867 dataset. PRKAR1A, SDCBP, SPRED2, and VAMP3 were confirmed to be upregulated in the two datasets. Furthermore, based on scRNA-seq data of the GSE135310 dataset, an autocrine/paracrine cross-talk network between monocytes/macrophages and neutrophils was identified in AMI mice. Upregulated PRKAR1A and SDCBP were found to be correlated with levels of the abovementioned two cell subpopulations. Increased L–R pair genes from monocytes/macrophages and neutrophils as well as these two genes might be involved in inflammatory response, apoptosis, and angiogenesis during the early phase of AMI. Finally, PRKAR1A and SDCBP were proved to be potential biomarkers for AMI patients who were at a high risk of HF progression.

In Niu et al.’s study (26), WGCNA was also applied to analyze the GSE59867 dataset, and 6 hub genes were detected, namely, BCL3, HCK, PPIF, S100A9, SERPINA1, and TBC1D9B, which were different from our study. The main reason for the discrepancy could be related to the different criteria and workflow of defining a potential hub gene between these two studies. Especially, the expression levels of hub genes were not verified in the datasets used for screening hub genes in Niu et al.’s study. We, in the present study, measured the expression levels of the hub genes in the GSE11947 and GSE59867 datasets. Among these 6 genes, the BCL3 expression level was significantly different in the GSE11947 dataset, rather than in the GSE59867 dataset. The TBC1D9B expression level was not detected in the GSE11947 dataset. Other genes were not statistically significant in the two datasets (**Supplementary Figure 6**). In our study, after screening hub genes, the expression levels of these genes were validated in both GSE11947 and GSE59867 datasets. Only hub genes, PRKAR1A, SDCBP, SPRED2, and VAMP3, showing consistent changes in the two datasets, were selected as candidate marker genes. It could be helpful to explore more reliable biomarkers by combining the WGCNA with expression levels of hub genes.

C-reactive protein (CRP) was first reported as a biomarker of HF (27). A great number of biomarkers were subsequently presented for HF, and the B-type natriuretic peptide (BNP) level was recognized as the diagnostic gold standard of HF (28). Nevertheless, the BNP level could not predict whether AMI patients with normal pressure of the left ventricle may suffer from HF during the follow-up. A cohort study reported that RNA levels of QSOX1 and PLBD1 in peripheral blood were potential predictors of left ventricular dysfunction after AMI (29). In the present study, the gene expression profiles of whole blood cells of patients within 12 h after AMI were analyzed using WGCNA, which provided a new direction to explore biomarkers for the early identification of AMI patients who were at a high risk of HF progression.

A tremendous loss of cardiomyocytes after AMI may result in adverse ventricular remodeling, including complicated changes in morphology, physiological functions, and cellular and molecular mechanisms of the ventricle (30). Irreversible left ventricular remodeling is similar to the wound healing response, which is characterized by myocardial cell apoptosis, inflammatory and immune responses, degradation of the extracellular matrix, myocardial hypertrophy, and fibrogenesis (31, 32). In the current study, functional enrichment analyses of genes in the post-HF-related module, namely, greenyellow module, identified by WGCNA, revealed that these hub genes were primarily involved in inflammation, immune response, and apoptosis, indicating a close relationship of the pathological progression of ventricular remodeling with HF (33–36).

Neutrophils, monocytes/macrophages, T lymphocytes, and B lymphocytes play important roles in myocardial inflammation and immune system activation after AMI (37, 38). Neutrophils are the first circulating innate immune cells to massively infiltrate the heart after myocardial infarction during the first 24–48 h after AMI, followed by the infiltration of monocytes, T lymphocytes, and B lymphocytes into the damaged site. Consistent with previous findings, at scRNA-seq levels, we identified that monocytes/macrophages, neutrophils, T lymphocytes, and B lymphocytes were involved in the early phase of AMI. Especially, it was identified that PRKAR1A and SDCBP were upregulated in whole blood cells (GSE11947 dataset) and PBMCs (GSE59867 dataset) within the first day of AMI patients who developed HF during the follow-up. Besides, the results of scRNA-seq further suggested that monocytes may be the major contributor to the upregulation of circulating PRKAR1A, whereas both neutrophils and monocytes were the contributors to the upregulation of circulating SDCBP. VAMP3 and SPRED2, which were validated in the GSE59867 and GSE11947 datasets while being not significantly upregulated in the GSE135310 dataset, were probably released by other cell types of leukocytes. Complicated intercellular communication mainly starts with the binding of a ligand to its corresponding receptor, followed by the activation of a specific signaling pathway. Identifying L–R pairs is important to understanding cellular behavior and to predicting the underlying mechanisms of cell-to-cell interactions (39). Through the cell–cell communication analysis, we found that upregulated L–R genes along with candidate marker genes (PRKAR1A and SDCBP) were mainly involved in inflammation, leukocyte proliferation, apoptosis, and angiogenesis. These biological processes are highly relevant to the pathological mechanism of myocardial injury and remolding after AMI.

In the present study, after the validation and ROC curve analysis based on three independent datasets, PRKAR1A and SDCBP were ultimately detected as potentially predictive biomarkers of post-AMI HF. PRKAR1A is highly expressed in the heart and controls PKA kinase activity by separating PKA catalytic subunits. A recent study revealed that the cardiac-specific ablation of PRKAR1A in mice reduced cardiomyocyte hypertrophy through PKA-dependent Drp1 inactivation (40). Additionally, PRKAR1A depletion protected cells against endoplasmic reticulum stress-induced apoptosis through the same molecular mechanism (16). Consistent with this observation, PRKAR1A overexpression in myocyte cells prompted oxidation-induced cell apoptosis and inhibited mitochondrial respiration (41). Collectively, these findings suggested that upregulation of PRKAR1A might contribute to the adverse cardiac remodeling. Syndecan-binding protein 1, also known as syntenin, encoded by the SDCBP gene, is a PDZ domain-containing adaptor that is involved in a variety of functions, including cytoskeletal-membrane organization, cell adhesion, trafficking of transmembrane proteins, immunomodulation, and tumorigenesis (42). SDCBP could modulate cancer cell motility and invasion through activating focal adhesion kinase (FAK), p38 MAPK, and NF-κB pathways (43). Moreover, SDCBP could positively regulate transforming growth factor beta-1 (TGF-β1)-mediated SMAD2/3 activation by preventing the caveolin-1-mediated internalization of the TGF-β type I receptor (44). Signaling pathways, such as p38 MAPK, NF-κB, and TGF-β pathways, were all implicated in the cardiac remodeling after AMI. Hence, SDCBP might exert an adverse effect on cardiac remodeling after AMI. In summary, the upregulation of circulating SDCBP and PRKAR1A in AMI patients with HF progression might modulate cardiac remodeling *via* a complex molecular regulatory network and serve as a potential therapeutic target of post-AMI HF.

Based on the results of the ROC curve analysis, AUC is considered as a valid measure to elucidate the overall diagnostic accuracy, and the AUC of 0.7–0.8 is acceptable, 0.8–0.9 is excellent, and more than 0.9 is prominent (45). The AUC values of PRKAR1A and SDCBP as well as the combination of these two genes in the GSE11947 and GSE59867 datasets were all more than 0.7, indicating a high diagnostic accuracy for the early diagnosis of AMI patients who were at a high risk of HF progression.

### Limitations

Firstly, the small sample size is a major limitation of the current study. To date, few studies have concentrated on the screening biomarkers for prediction of HF post-AMI based on gene-expression profiling; thus, only two human datasets were included in this study. Secondly, the expression profiles of the GSE11947 and GSE59867 datasets were generated from microarray. Microarray is commonly utilized, while RNA-seq is more sensitive to detect low abundance transcripts and enables the differentiation between isoforms and the identification of gene variants compared with microarray (46). Finally, in the GSE11947 dataset, the key data, such as the EF value or NT-proBNP level, were not available at the time of the blood collection within 12 h after AMI, which could influence the accuracy of screened genes as predictive biomarkers of HF post-AMI if some HF patients had already reduced EF on admission. It is therefore essential to collect enough blood samples to evaluate the predictive value of PRKAR1A and SDCBP for post-AMI HF in the future studies.

## Conclusions

In the current study, we applied bioinformatics analyses to identify potential biomarkers for AMI patients who were at a high risk of HF progression, and two genes, PRKAR1A and SDCBP, were screened due to their consistent expression changes in the study group and validation group. These two genes were considered as promising prognostic biomarkers and may also become potential therapeutic targets of AMI-triggered cardiac remodeling. Larger clinical trials are therefore required to further validate the predictive value of these two genes as biomarkers of HF progression after AMI before clinical application.

## Data Availability Statement

The datasets presented in this study can be found in online repositories. The names of the repository/repositories and accession number(s) can be found in the article/**Supplementary Material**.

## Ethics Statement

In the GSE11947 dataset, the study was approved by the local ethics committee (Comité National d’Ethique de la Recherché, CNER), and written informed consent was obtained from all patients. In the GSE59867 dataset, the study was approved by the local Ethics Committees of the Medical University of Warsaw and Medical University of Bialystok and was conducted in accordance with the principles of the Declaration of Helsinki. All participants gave written informed consent. In the GSE135310 dataset, all animal studies and numbers of animals used conform to the Directive 2010/63/EU of the European Parliament and have been approved by the appropriate local authorities (Regierung von Unterfranken, Würzburg, Germany, Akt.-Z. 55.2-DMS-2532-2-743 2 and Akt.-Z. 55.2-DMS-2532-2-865). Exclusion criteria related to general animal health had been defined upon preregistration of the experiments for approval by local authorities.

## Author Contributions

SL, QC, and HC designed and supervised the study. QC. analyzed the data and drafted the manuscript. LS searched the literatures. CL prepared the figures and tables. FG and QY revised the manuscript. All authors contributed to the article and approved the submitted version.

## Funding

This research was funded by the Peking University Medicine Seed Fund for Interdisciplinary Research (BMU2022MX009), the Peking University Medicine Fund of Fostering Young Scholars’ Scientific & Technological Innovation (BMU2021PY009), the National Natural Science Foundation of China (No. 81970301, No. 81600340), the Beijing Municipal Natural Science Foundation (No. 7202218), and the Capital Health Research and Development of Special (No. 2020-2-4084).

## Conflict of Interest

The authors declare that the research was conducted in the absence of any commercial or financial relationships that could be construed as a potential conflict of interest.

## Publisher’s Note

All claims expressed in this article are solely those of the authors and do not necessarily represent those of their affiliated organizations, or those of the publisher, the editors and the reviewers. Any product that may be evaluated in this article, or claim that may be made by its manufacturer, is not guaranteed or endorsed by the publisher.

## Supplementary Material

Supplementary Table 1 Demographic characteristics of post-AMI HF (n = 9) and non-HF (n = 8) patients from dataset GSE59867.

Supplementary Table 2 Genes of greenyellow module.

Supplementary Table 3 GO biological processes in greenyellow module.

Supplementary Table 4 Pathways analyses in greenyellow module.

Supplementary Table 5 Hub genes of greenyellow module.

Supplementary Table 6 Overlapped genes between GSE11947 and GSE59867.

Supplementary Table 7 Markers of Monocytes/Macrophages 2 days after AMI and Markers of Monocytes/Macrophages and Neutrophils 3 days after AMI.

Supplementary Table 8 Up-regulated L-R pairs of Monocytes/Macrophages and Neutrophils base on cell-cell communication analysis.

Supplementary Table 9 GO biological process annotation of PRKAR1A and significant L-R pairs genes in Monocytes/Macrophages (post-AMI 2 days).

Supplementary Table 10 GO biological process annotation of SDCBP and significant L-R pairs genes in Neutrophils (post-AMI 2 days).

Supplementary Table 11 GO biological process annotation of SDSBP and significant L-R pairs genes in Monocytes/Macrophages (post-AMI 3 days).

Supplementary Figure 1 Identification of modules using WGCNA algorithm.

Supplementary Figure 2 The Clustering dendrogram of modules eigengenes and heatmaps of the correlations between eigengenes.

Supplementary Figure 3 Functional enrichment analyses of genes in greenyellow module through Metascape.

Supplementary Figure 4 Scatter plot of greenyellow module eigengenes.

Supplementary Figure 5 Analysis of scRNA seq dataset GSE135310 and cell type classification analysis.

Supplementary Figure 6 Validation of the expression levels of real hubgenes of Niu’s study in post-AMI HF and post-AMI non-HF patients based on datasets GSE11947 and GSE59867.

## Abbreviations

AMI, acute myocardial infarction; HF, heart failure; WGCNA, weighted gene co-expression network analysis; scRNA-seq, single-cell RNA sequencing; SS, steady state; Mon/Mac, monocytes/macrophages; Neu, neutrophils; L–R pairs, ligand–receptor pairs; NPs, natriuretic peptides; sST2, soluble suppression of tumorigenicity-2; DEGs, differentially expressed genes; ROC, receiver operating characteristic; EF, ejection fraction; GO, gene ontology; bp, biological processes; KEGG, Kyoto Encyclopedia of Genes and Genomes; GS, gene significance; MM, module membership; cor, correlation coefficient; Mon/Mac, monocytes/macrophages; PRKAR1A, protein kinase cAMP-dependent type I regulatory subunit alpha; SDCBP, Syndecan-binding protein 1/Syntenin; SPRED2, EVH1-domain-containing protein 2; VAMP3, vesicle-associated membrane protein-3; AUC, area under the ROC curve; CRP, C-reactive protein; BNPs, B-type natriuretic peptides; MAD, median absolute deviation; TOM, topological overlap matrix; MEs, module eigengenes; SEM, standard error of mean
