# Supplementary material for: PRKAR1A and SDCBP Serve as Potential Predictors of Heart Failure Following Acute Myocardial Infarction
Source: Front Immunol. 2022 May 3;13:878876. doi: 10.3389/fimmu.2022.878876 (PMC9110666; doi:10.3389/fimmu.2022.878876)
Supplement: Supplementary Table 1 — Demographic characteristics of post-AMI HF (n = 9) and non-HF (n = 8) patients from dataset GSE59867. [file Table_1.pdf]

TABLE 1. Baseline demographic and clinical characteristics of HF, non-HF patients from dataset GSE59867

**Characteristics**	**HF patients (n = 9)**	**Non-HF patients (n = 8)**	***P* value**
Men	6 (66.7%)	7 (87.5%)	0.576
Women	3 (33.3%)	1 (12.5%)	0.576
Age (years)	60.1 ± 14.3	51.8 ± 7.2	0.147
BMI (kg/m^2)	26.8 ± 3.1	25.6 ± 1.6	0.323
Hypertension	3 (33.3%)	1 (12.5%)	0.576
Diabetes	2 (22.2%)	1 (12.5%)	>0.999
Previous MI	0 (0%)	0 (0%)	NA
Smoking	3 (33.3%)	5 (62.5%)	0.347
Hypercholesterolemia	5 (55.6%)	4 (50%)	>0.999
AMI	8 (88.9%)	3 (42.9%)	0.106
NT-proBNP (pg/mL)[a]	918.3 ± 848.5	62 ± 14.1	<0.001
LVEF (%)[a]	39.3 ± 8.4	66.8 ± 1.9	0.001
Medications			
Aspirin	9 (100%)	8 (100%)	NA
Clopidogrel	8 (88.9%)	8 (100%)	>0.999
Beta blockers	9 (100%)	8 (100%)	NA
ACE inhibitors	9 (100%)	8 (100%)	NA
Statins	9 (100%)	8 (100%)	NA
Diuretics	7 (77.8%)	1 (12.5%)	0.015

[a]NT-proBNP, LVEF measured 6 months after AMI.

Data are presented as mean value ± standard deviation or number or percentage of patients. P value <0.05 was considered significant.

ACE, angiotensin-converting enzyme; AMI, anterior myocardial infarction; BMI, body mass index; LVEF, left ventricular ejection fraction; MI, myocardial infarction; NA, not applicable; NT-proBNP, N-terminal pro-brain natriuretic peptide.
