# Supplementary material for: PRKAR1A and SDCBP Serve as Potential Predictors of Heart Failure Following Acute Myocardial Infarction
Source: Front Immunol. 2022 May 3;13:878876. doi: 10.3389/fimmu.2022.878876 (PMC9110666; doi:10.3389/fimmu.2022.878876)
Supplement: Supplementary Table 2 — Genes of greenyellow module. [file Table_2.pdf]

TABLE 2. Genes of greenyellow module

Gene symbol	Module
FACL2;ACSL1	greenyellow
SDCBP	greenyellow
PRKAR1A	greenyellow
SRP9	greenyellow
CEBPB	greenyellow
SAMSN1	greenyellow
SLC38A2	greenyellow
PRKDC	greenyellow
C6ORF209	greenyellow
TXNRD1	greenyellow
RALB	greenyellow
MCL1	greenyellow
CAPZA2	greenyellow
ZFP36L2	greenyellow
LOC91496	greenyellow
ASAH1	greenyellow
ME2	greenyellow
PSAP	greenyellow
DNAJC7	greenyellow
PP784	greenyellow
SDFR1	greenyellow
M17S2	greenyellow
CDC10	greenyellow
TXNIP	greenyellow
ZA20D3	greenyellow
ANP32C	greenyellow
GDI2	greenyellow
HIRA	greenyellow
K-ALPHA-1;TUBA3	greenyellow
PICALM;AF10/CALM	greenyellow
MI-ER1	greenyellow
VAMP3	greenyellow
DNAJB6	greenyellow
CROP	greenyellow
C9orf10	greenyellow
CCNG1	greenyellow
RAD21	greenyellow
ZNF217	greenyellow
IL8	greenyellow
SNAP23	greenyellow
RHOT1	greenyellow
JAK1	greenyellow
MGC11061	greenyellow
TEX27	greenyellow

ELOVL5	greenyellow
SSFA2	greenyellow
PYGL	greenyellow
HSPDP3;HSPDP1	greenyellow
PPP2CB	greenyellow
ARRDC3	greenyellow
TPM3;OPLAH	greenyellow
MGC33864	greenyellow
HLA-E	greenyellow
TAF7	greenyellow
GALNT7	greenyellow
THRAP2	greenyellow
RAB1A	greenyellow
RAB5A	greenyellow
LRRFIP1	greenyellow
TOB1	greenyellow
PLSCR1	greenyellow
HSGP25L2G	greenyellow
ELF1	greenyellow
WDFY1	greenyellow
SLC7A5	greenyellow
ZCCHC2	greenyellow
PSMD10	greenyellow
SERP1	greenyellow
KDELR3	greenyellow
GHITM	greenyellow
SCOC	greenyellow
TYR	greenyellow
MAML1	greenyellow
ADP-GK	greenyellow
MAT2B	greenyellow
RNF6	greenyellow
RAP1A	greenyellow
UTX	greenyellow
CRSP3	greenyellow
DDX55	greenyellow
SMAP-5	greenyellow
SIAH1	greenyellow
FLJ12716	greenyellow
FLJ13576	greenyellow
C6ORF109	greenyellow
SORL1	greenyellow
C15ORF23	greenyellow
DCK	greenyellow
SMNDC1	greenyellow
FBXO38	greenyellow

BEX1	greenyellow
MRPL44	greenyellow
FLJ12666	greenyellow
RNF11	greenyellow
TLK2	greenyellow
CRK	greenyellow
CNAP1	greenyellow
VRK2	greenyellow
SPTLC2	greenyellow
ETS2	greenyellow
PPM1B	greenyellow
CSAD	greenyellow
LOC120224	greenyellow
UBC	greenyellow
BTEB1	greenyellow
SPTBN1	greenyellow
SDC2	greenyellow
SEP01;XRN1	greenyellow
POLR2C	greenyellow
SC5DL	greenyellow
GGH	greenyellow
PAI1	greenyellow
KIAA1012	greenyellow
SYAP1	greenyellow
UBE2M	greenyellow
NUP153	greenyellow
FEN1	greenyellow
DKFZp586M1819	greenyellow
PSMD11	greenyellow
KIAA1026	greenyellow
FLJ13910	greenyellow
GNB1	greenyellow
EML2	greenyellow
C14ORF154	greenyellow
TMEM2	greenyellow
MSL3L1	greenyellow
DKFZP586L0724	greenyellow
PHF14	greenyellow
ZBED4	greenyellow
TA-PP2C	greenyellow
SIAT4C	greenyellow
NIPBL	greenyellow
SPRED2	greenyellow
NDRG3	greenyellow
DAP	greenyellow
MGC71745	greenyellow

KIAA0891	greenyellow
KIAA1271	greenyellow
TOMM34	greenyellow
LOC143308	greenyellow
SEC24D	greenyellow
TOR3A	greenyellow
PTK9L	greenyellow
ABI1	greenyellow
ADAM10	greenyellow
DHTKD1	greenyellow
RPS6KA2	greenyellow
LAPTM4B	greenyellow
KIAA1010	greenyellow
QSCN6	greenyellow
ENSA	greenyellow
VPS41	greenyellow
TDE1	greenyellow
ERP70	greenyellow
LOC283929	greenyellow
FLJ32205	greenyellow
ZWINT	greenyellow
ARL6IP	greenyellow
FLJ14753	greenyellow
GGPS1	greenyellow
NOTCH2	greenyellow
LOC387680	greenyellow
CCM1	greenyellow
ARHGAP12	greenyellow
LOC283120	greenyellow
CHKA	greenyellow
ATF1	greenyellow
CGI-09	greenyellow
MAML3	greenyellow
ENTH	greenyellow
ALDOC	greenyellow
LOC220213	greenyellow
MGC22265;SMA3;LOC153	greenyellow
FLJ00254	greenyellow
ROCK1	greenyellow
SEH1L	greenyellow
PIAS1	greenyellow
TMEM23	greenyellow
TRRAP	greenyellow
BCL2L2	greenyellow
UBXD2	greenyellow
EPS15	greenyellow

ASB8	greenyellow
CTHRC1	greenyellow
MFAP3	greenyellow
FLJ39378	greenyellow
LOC56930	greenyellow
CDCA5	greenyellow
PIK3CB	greenyellow
RANBP2L1;GCC2	greenyellow
FLJ32642	greenyellow
LOC133308	greenyellow
HMG2L1	greenyellow
CBS	greenyellow
MTRR	greenyellow
SENP2	greenyellow
KIAA0401	greenyellow
ANKRA2	greenyellow
#NA;SYT7	greenyellow
#NA;MYLK	greenyellow
CRADD	greenyellow
TAFA5	greenyellow
PPM1G	greenyellow
TCEAL1	greenyellow
PPFIA1	greenyellow
TNNT3	greenyellow
AKR1D1	greenyellow
FLJ25416	greenyellow
LOC51249	greenyellow
PTER	greenyellow
PLA2G6	greenyellow
RGS7	greenyellow
FLJ11082	greenyellow
YBX2	greenyellow
ANXA9	greenyellow
JRK	greenyellow
LOC219953	greenyellow
MAP4K3	greenyellow
