# Supplementary material for: PRKAR1A and SDCBP Serve as Potential Predictors of Heart Failure Following Acute Myocardial Infarction
Source: Front Immunol. 2022 May 3;13:878876. doi: 10.3389/fimmu.2022.878876 (PMC9110666; doi:10.3389/fimmu.2022.878876)
Supplement: Supplementary Table 4 — Pathways analyses in greenyellow module. [file Table_4.pdf]

TABLE 4. Pathways in greenyellow module

Term	Description	Gene counts	Percent	Log P	Log(Q-value)
**Greenyellow**					
R-HSA-199991	Membrane trafficking	20	0.03	-7.58	-4.10
R-HSA-68886	M phase	16	0.04	-7.29	-4.10
R-HSA-9013694	Signaling by NOTCH4	7	0.09	-5.73	-3.15
R-HSA-2262752	Cellular responses to stress	16	0.03	-5.37	-2.92
R-HSA-9006934	Signaling by receptor tyrosine kinases	14	0.03	-4.81	-2.51
R-HSA-187037	Signaling by NTRK1 (TRKA)	7	0.06	-4.75	-2.47
R-HSA-556833	Metabolism of lipids	17	0.02	-4.68	-2.45
R-HSA-5663202	Diseases of signal transduction by growth factor receptors and second messengers	12	0.03	-4.68	-2.45
R-HSA-6798695	Neutrophil degranulation	13	0.03	-4.41	-2.28
R-HSA-70171	Glycolysis	5	0.07	-3.83	-1.84
M161	IFNG pathway	4	0.10	-3.75	-1.81
ko04071	Sphingolipid signaling pathway	6	0.08	-3.71	-1.78
R-HSA-71291	Metabolism of amino acids and derivatives	10	0.08	-3.47	-1.64
R-HSA-6811436	COPI-independent Golgi-to-ER retrograde traffic	4	0.08	-3.34	-1.57
M252	IL8 CXCR1 pathway	3	0.11	-3.01	-1.38
M240	Syndecan2 pathway	3	0.09	-2.80	-1.22
M277	Intergrin A4B1 pathway	3	0.09	-2.80	-1.22
M16801	Regulation of the actin cytoskeleton by RHO GTPases	3	0.09	-2.72	-1.18
M229	P38 alpha beta downstream pathway	3	0.08	-2.62	-1.13
M145	P53 downstream pathway	5	0.04	-2.54	-1.09

KEGG: Kyoto Encyclopedia of Genes and Genomes.
