# Supplementary material for: PRKAR1A and SDCBP Serve as Potential Predictors of Heart Failure Following Acute Myocardial Infarction
Source: Front Immunol. 2022 May 3;13:878876. doi: 10.3389/fimmu.2022.878876 (PMC9110666; doi:10.3389/fimmu.2022.878876)
Supplement: Supplementary Table 5 — Hub genes of greenyellow module. [file Table_5.pdf]

TABLE 5. Hub genes of greenyellow module

Gene symbol	geneModuleMembership	geneTraitCor
SDCBP	0.850445675	0.416263
PRKAR1A	0.901884214	0.555889
SRP9	0.868881544	0.305387
SLC38A2	0.86423578	0.319872
PRKDC	0.914542918	0.480940
C6ORF209	0.84759081	0.393644
TXNRD1	0.90142534	0.348836
CAPZA2	0.865578877	0.437517
LOC91496	0.811547801	0.353879
ME2	0.846595561	0.379356
DNAJC7	0.823250003	0.327126
PP784	0.87665436	0.311264
SDFR1	0.882644638	0.404538
M17S2	0.880129358	0.323296
CDC10	0.877274227	0.284403
ZA20D3	0.847412927	0.427865
GDI2	0.815696602	0.265642
PICALM;AF10/CALM	0.852301481	0.461983
MI-ER1	0.817628805	0.335921
VAMP3	0.909260447	0.481449
DNAJB6	0.910167003	0.332858
CROP	0.899853149	0.316238
CCNG1	0.834388009	0.207055
RAD21	0.916750835	0.360042
ZNF217	0.866049269	0.451263
SNAP23	0.845489823	0.387725
RHOT1	0.913401153	0.485725
MGC11061	0.893280059	0.460991
SSFA2	0.910894408	0.476973
PPP2CB	0.906815656	0.445367
TPM3;OPLAH	0.877145316	0.431985
MGC33864	0.884220875	0.454911
TAF7	0.844821569	0.285458
GALNT7	0.944413223	0.490767
RAB1A	0.822243793	0.436159
RAB5A	0.862256439	0.397827
TOB1	0.814785477	0.584559
WDFY1	0.80969593	0.234844
PSMD10	0.831169617	0.459408
SERP1	0.882109837	0.379817
SCOC	0.848543011	0.466862
MAT2B	0.889779618	0.302821
RNF6	0.842973999	0.243164
CRSP3	0.853891897	0.252158

SIAH1	0.871187738	0.369621
FLJ13576	0.880643181	0.276002
C15ORF23	0.864516273	0.484134
DCK	0.85473933	0.396370
SMNDC1	0.928265853	0.363401
MRPL44	0.849553359	0.314007
FLJ12666	0.825613198	0.476089
TLK2	0.920851362	0.416243
VRK2	0.842598418	0.329766
PPM1B	0.809241573	0.416094
CSAD	0.826318639	0.451215
BTEB1	0.831265353	0.353437
SPTBN1	0.850622581	0.380737
PAI1	0.816662625	0.405593
KIAA1012	0.922947785	0.422583
NUP153	0.820876159	0.387946
C14ORF154	0.90225582	0.359818
TMEM2	0.852140012	0.347765
ZBED4	0.868815259	0.373607
SPRED2	0.825839122	0.436853
ARHGAP12	0.81520977	0.318954
PIAS1	0.800171476	0.500161
MTRR	0.814214647	0.516151
AKR1D1	0.817583734	0.577070
