# Supplementary material for: PRKAR1A and SDCBP Serve as Potential Predictors of Heart Failure Following Acute Myocardial Infarction
Source: Front Immunol. 2022 May 3;13:878876. doi: 10.3389/fimmu.2022.878876 (PMC9110666; doi:10.3389/fimmu.2022.878876)
Supplement: Supplementary Table 6 — Overlapped genes between GSE11947 and GSE59867. [file Table_6.pdf]

TABLE 6. Overlapped genes between GSE11947 and GSE59867

Gene symobol	Module
SRP9	greenyellow
RAB5A	greenyellow
SDCBP	greenyellow
DCK	greenyellow
RAB1A	greenyellow
RAD21	greenyellow
VAMP3	greenyellow
SPTBN1	greenyellow
PSMD10	greenyellow
SMNDC1	greenyellow
ZNF217	greenyellow
SLC38A2	greenyellow
ME2	greenyellow
TXNRD1	greenyellow
TAF7	greenyellow
SERP1	greenyellow
NUP153	greenyellow
ARHGAP12	greenyellow
SPRED2	greenyellow
CSAD	greenyellow
TMEM2	greenyellow
PRKDC	greenyellow
ZBED4	greenyellow
CAPZA2	greenyellow
SIAH1	greenyellow
CCNG1	greenyellow
SCOC	greenyellow
MTRR	greenyellow
MAT2B	greenyellow
AKR1D1	greenyellow
GDI2	greenyellow
PPM1B	greenyellow
DNAJC7	greenyellow
DNAJB6	greenyellow
VRK2	greenyellow
TLK2	greenyellow
SNAP23	greenyellow
GALNT7	greenyellow
SSFA2	greenyellow
TOB1	greenyellow
WDFY1	greenyellow
RHOT1	greenyellow
PRKAR1A	greenyellow
MRPL44	greenyellow

PPP2CB	greenyellow
RNF6	greenyellow
PIAS1	greenyellow
