# Supplementary material for: PRKAR1A and SDCBP Serve as Potential Predictors of Heart Failure Following Acute Myocardial Infarction
Source: Front Immunol. 2022 May 3;13:878876. doi: 10.3389/fimmu.2022.878876 (PMC9110666; doi:10.3389/fimmu.2022.878876)
Supplement: Supplementary Table 9 — GO biological process annotation of PRKAR1A and significant L-R pairs genes in Monocytes/Macrophages (post-AMI 2 days). [file Table_9.pdf]

Table 9. GO biological process annotation of Prkar1a and significant L-R pairs genes in Monocytes/Macrophages (post-AMI 2 days).

Term	Description	Log(Q-value)	Gene
GO:0070661	leukocyte proliferation	-17.03	*Cd44, Cxcr4, Ccr2, Csf1, Csf1r, Cd74, Itgam, Itgb2, Lgals9, Mif,* ***Prkar1a****, Ptprc, Ccl12, Ccl5, Tnfrsf1b, Havcr2, Ccr5, Itga5, Itgb1, Ccl2, Spp1, Tnf, C3, Ccr1, C3ar1*
GO:0045123	cellular extravasation	-8.44	*Ccr2, Itgam, Itgb1, Itgb2, Ccl2, Ccl5, Tnf, Cd44, Itga5, Ptprc, Csf1, Spp1, C3, Csf1r, Cxcr4,* ***Prkar1a****, Havcr2*
GO:0002699	positive regulation of immune effector process	-7.44	*C3, Ccr2, Cd74, Itgam, Itgb2, Mif, Ptprc, Ccl2, Tnf, Itgb1, Lgals9, Ccl5, Ccr1, Ccl12, Havcr2,* ***Prkar1a****, Cd44*
GO:2000343	positive regulation of chemokine (C-X-C motif) ligand 2 production	-6.55	*Cd74, Mif, Ccl5, Tnf, C3, Ccr5, Cxcr4, Ccr2, C3ar1, Nampt,* ***Prkar1a****, Ccl12, Lgals9, Ptprc, Spp1*

GO: Gene Ontology.
