# Supplementary material for: PRKAR1A and SDCBP Serve as Potential Predictors of Heart Failure Following Acute Myocardial Infarction
Source: Front Immunol. 2022 May 3;13:878876. doi: 10.3389/fimmu.2022.878876 (PMC9110666; doi:10.3389/fimmu.2022.878876)
Supplement: Supplementary Table 10 — GO biological process annotation of SDCBP and significant L-R pairs genes in Neutrophils (post-AMI 2 days). [file Table_10.pdf]

Table 10. GO biological process annotation of Sdcbp and significant L-R pairs genes in Neutrophils (post-AMI 2 days).

Term	Description	Log(Q-value)	Gene
GO:0002685	regulation of leukocyte migration	-12.282	*Cxcr2, Ccr1, Csf1, Csf1r, Cd74, Il1a, Il1b, Lgals9, Anxa1, Mif, Ccl7, Cxcl1, Ccl3, Tnf, Ptprc,* ***Sdcbp***
GO:0032944	regulation of mononuclear cell proliferation	-11.890	*Cd44, Csf1, Csf1r, Cd74, Il1a, Il1b, Lgals9, Anxa1, Mif, Ptprc, Tnfrsf1b, Tnf, Il1r2, Ccl3, Fpr2, Fpr1, Cxcl1, Ccr1,* ***Sdcbp***
GO:1902107	positive regulation of leukocyte differentiation	-9.640	*Ccr1, Csf1, Csf1r, Cd74, Lgals9, Anxa1, Ptprc, Ccl3, Tnf, Cd44, Il1a, Il1b, Mif,* ***Sdcbp***
GO:0032722	positive regulation of chemokine production	-8.785	*Csf1r, Cd74, Il1a, Il1b, Lgals9, Mif, Tnf, Cd44, Ccr1, Cxcl1, Anxa1, Ccl3, Tnfrsf1b, Cxcl2, Cxcr2, Ptprc, Csf1, Il1r2, Ccl9,* ***Sdcbp***
GO:0033032	regulation of myeloid cell apoptotic process	-5.085	*Cd44, Cxcr2, Anxa1, Mif, Il1b, Il1r2, Tnf, Tnfrsf1b,* ***Sdcbp****, Csf1, Il1a, Csf1r*

GO: Gene Ontology.
