# Supplementary material for: PRKAR1A and SDCBP Serve as Potential Predictors of Heart Failure Following Acute Myocardial Infarction
Source: Front Immunol. 2022 May 3;13:878876. doi: 10.3389/fimmu.2022.878876 (PMC9110666; doi:10.3389/fimmu.2022.878876)
Supplement: Supplementary Table 11 — GO biological process annotation of SDSBP and significant L-R pairs genes in Monocytes/Macrophages (post-AMI 3 days). [file Table_11.pdf]

Table 11. GO biological process annotation of Sdcbp and significant L-R pairs genes in Monocytes/Macrophages (post-AMI 3 days).

Term	Description	Log(Q-value)	Gene
GO:0002688	regulation of leukocyte chemotaxis	−11.039	*Ccr1, Ccr2, Cd74, Lgals9, Mif, Ccl12, Ccl2, Ccl5, Ccl7, Cxcr4, Itga5, Itgav, Itgb1,* ***Sdcbp****, Ccl8*
GO:0045785	positive regulation of cell adhesion	−10.478	*Cd44, Ccr2, Ccr5, Cd74, Itga5, Itgav, Itgb1, Lgals9, Ccl2, Ccl5, Spp1, Tnf, Cxcr4, Mif, Ccl12, Tnfrsf1b, Tnfrsf1a,* ***Sdcbp***
GO:0048245	eosinophil chemotaxis	−8.180	*Ccl12, Ccl2, Ccl5, Ccl7, Ccl8, Ccr2, Mif, Ccl3, Tnf, Itgav, Lgals9, Tnfrsf1b, Cxcr4, Ccr1, Itgb1, Ccr5, Cd74, Cd44, Spp1,* ***Sdcbp***
GO:0001525	angiogenesis	−7.113	*Cxcr4, Ccr2, Itga5, Itgav, Itgb1, Ccl12, Ccl2, Ccl5, Tnf, Tnfrsf1a, Cd44, Ccl3, Tnfrsf1b, Ccr5, Lgals9,* ***Sdcbp***
GO:0001934	positive regulation of protein phosphorylation	−6.980	*Cd44, Cxcr4, Cd74, Itga5, Itgb1, Lgals9, Mif, Ccl5, Tnf, Tnfrsf1a,* ***Sdcbp****, Tnfrsf1b, Ccr2, Ccr5, Itgav, Ccl2, Ccr1, Nampt*
GO:0034113	heterotypic cell-cell adhesion	−5.987	*Cd44, Itga5, Itgav, Itgb1, Tnf, Cxcr4, Ccr2, Ccr5, Ccl5, Spp1, Tnfrsf1a, Tnfrsf1b,* ***Sdcbp***
GO:0016049	cell growth	−1.736	*Cd44, Cxcr4, Ccr5, Itgb1,* ***Sdcbp***
GO:0032368	regulation of lipid transport	−1.457	*Itgav, Mif, Spp1, Tnf,* ***Sdcbp***
GO:0009895	negative regulation of catabolic process	−0.663	*Tnf,* ***Sdcbp****, Nampt, Cd44, Tnfrsf1b*

GO: Gene Ontology.
