# Supplementary figures and images for: PRKAR1A and SDCBP Serve as Potential Predictors of Heart Failure Following Acute Myocardial Infarction
Source: Front Immunol. 2022 May 3;13:878876. doi: 10.3389/fimmu.2022.878876 (PMC9110666; doi:10.3389/fimmu.2022.878876)

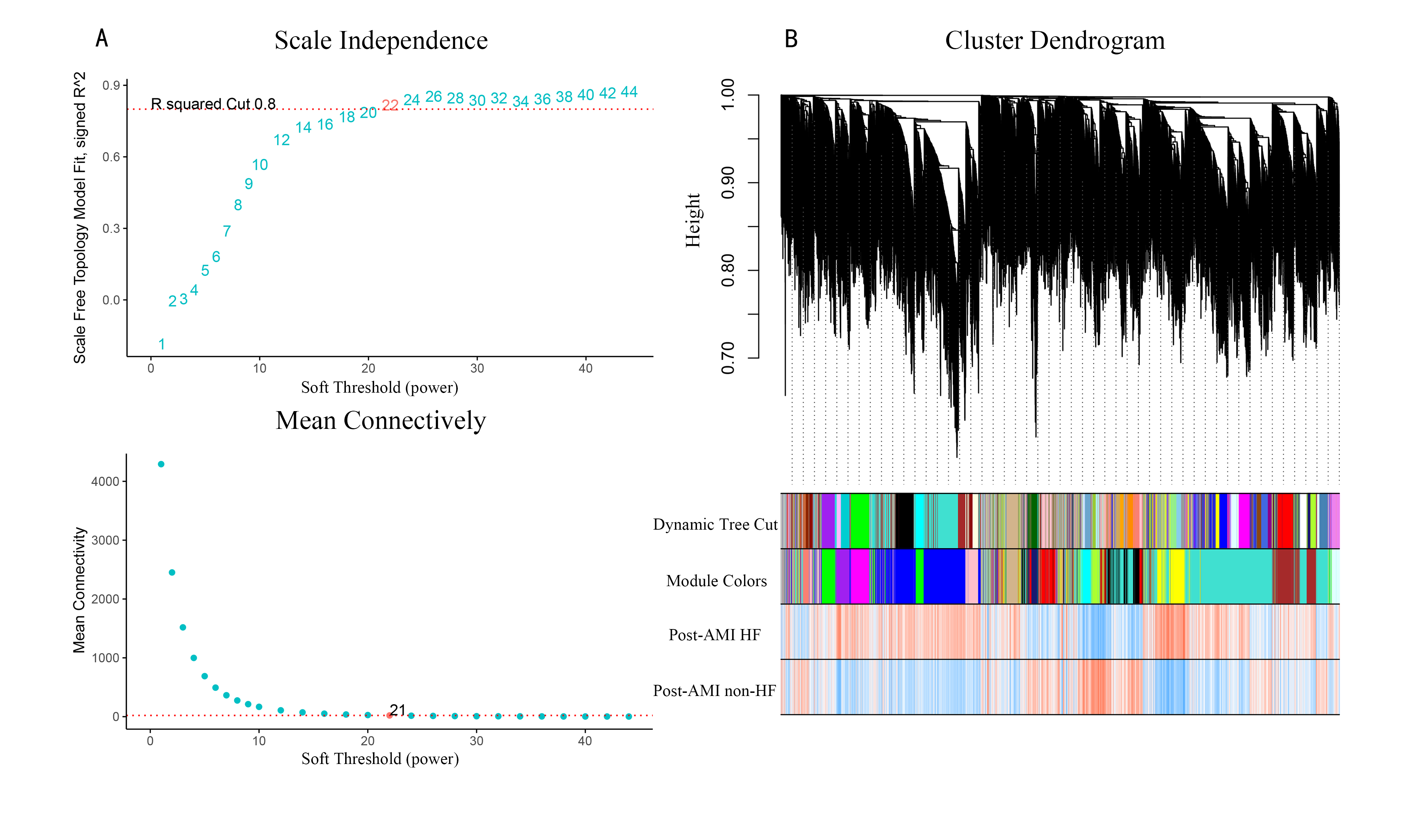

Supplement: Supplementary Figure 1 — Identification of modules using WGCNA algorithm. [file Image_1.tif]

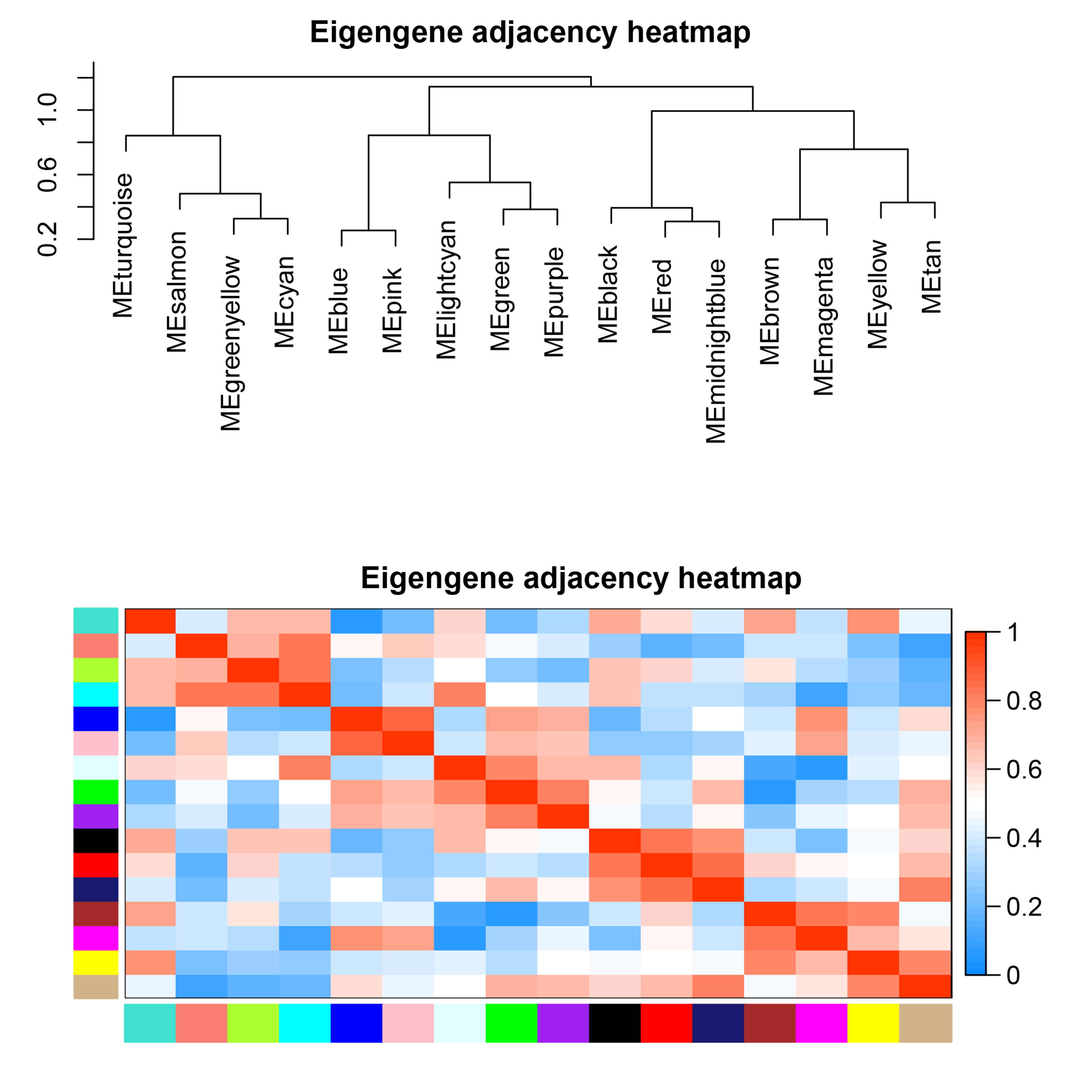

Supplement: Supplementary Figure 2 — The Clustering dendrogram of modules eigengenes and heatmaps of the correlations between eigengenes. [file Image_2.tif]

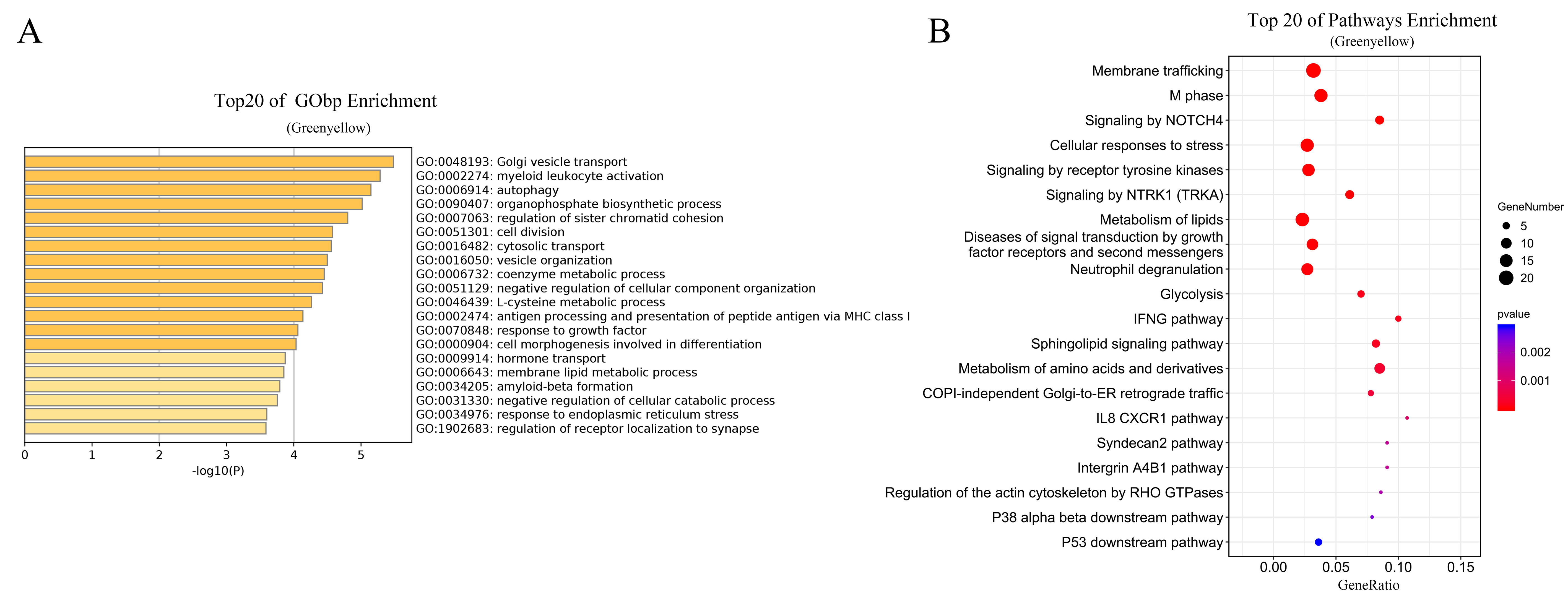

Supplement: Supplementary Figure 3 — Functional enrichment analyses of genes in greenyellow module through Metascape. [file Image_3.tif]

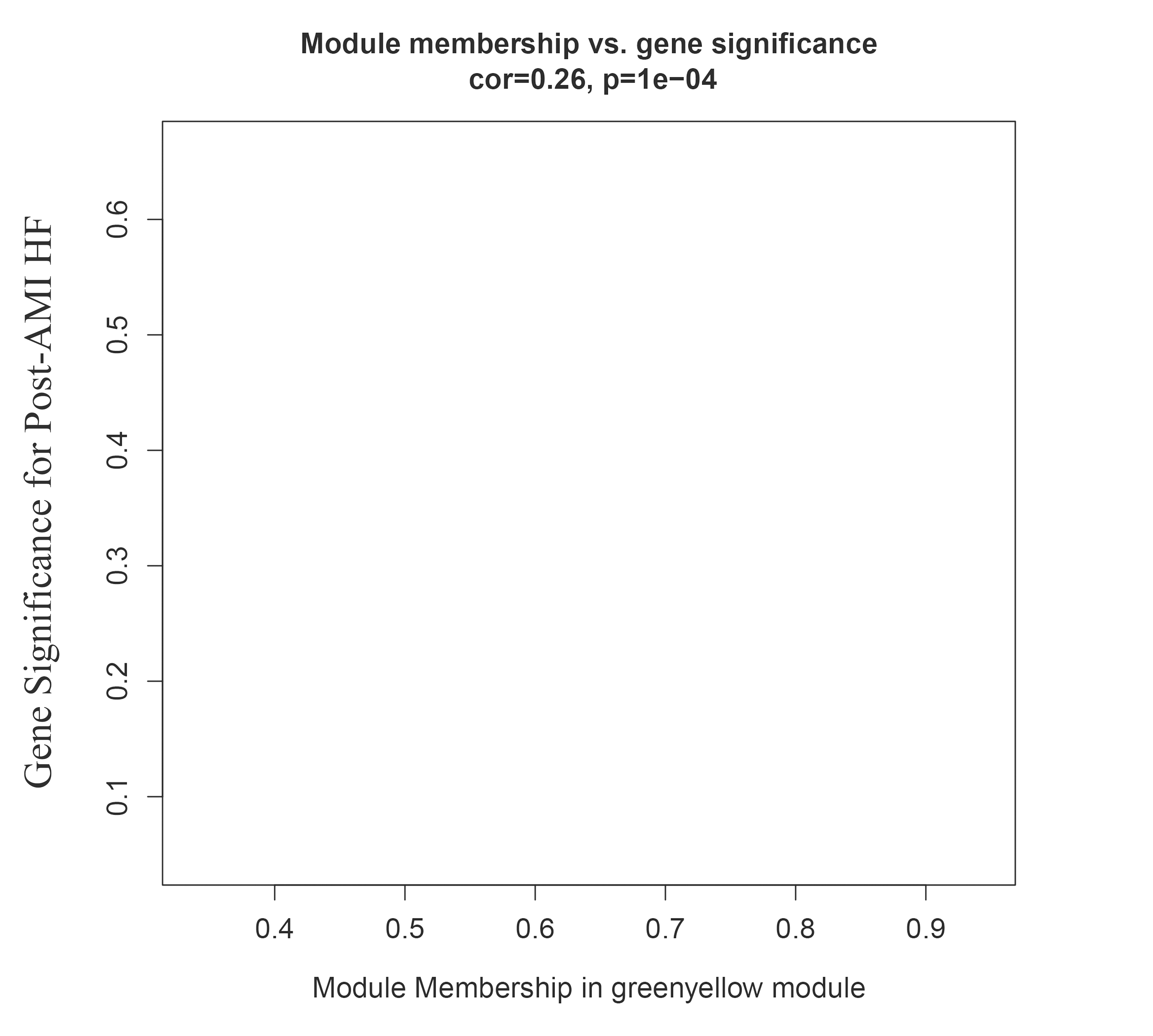

Supplement: Supplementary Figure 4 — Scatter plot of greenyellow module eigengenes. [file Image_4.tif]

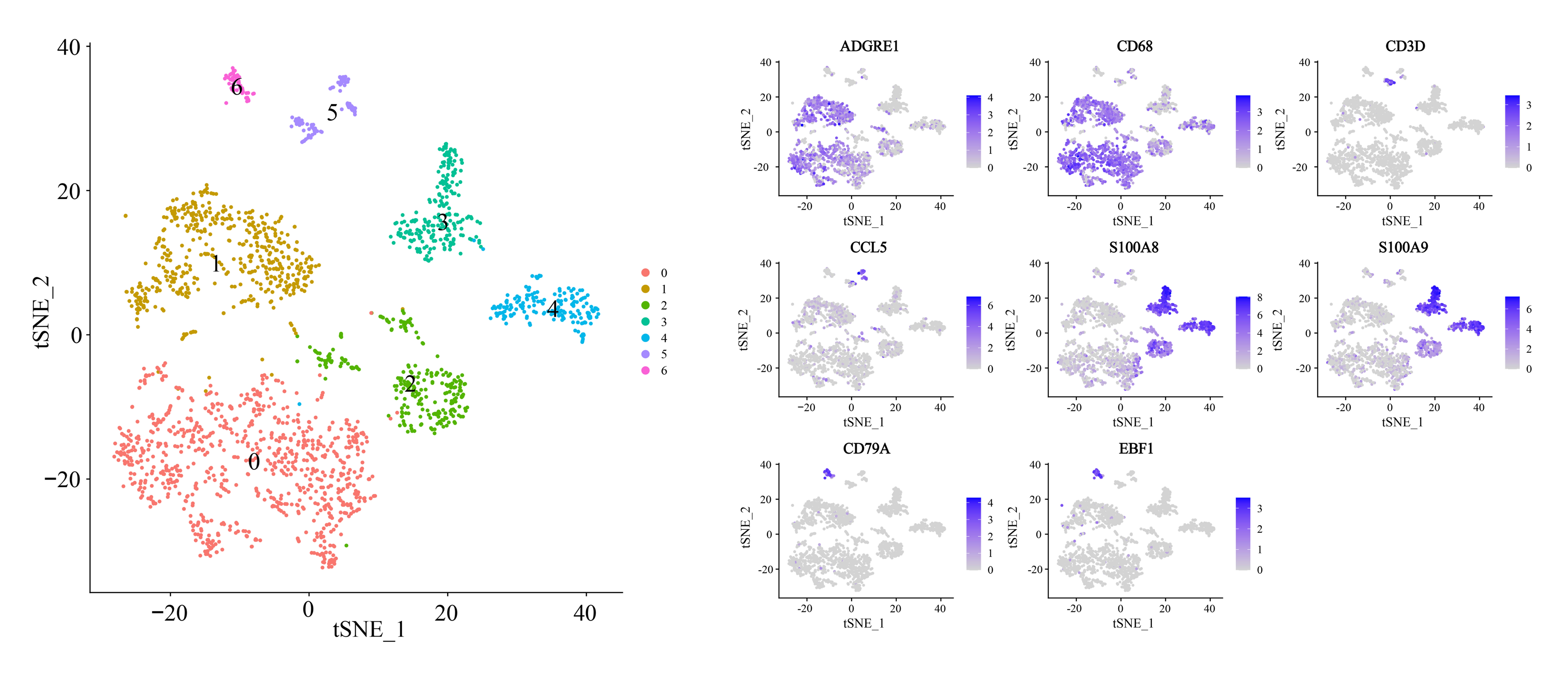

Supplement: Supplementary Figure 5 — Analysis of scRNA seq dataset GSE135310 and cell type classification analysis. [file Image_5.tif]

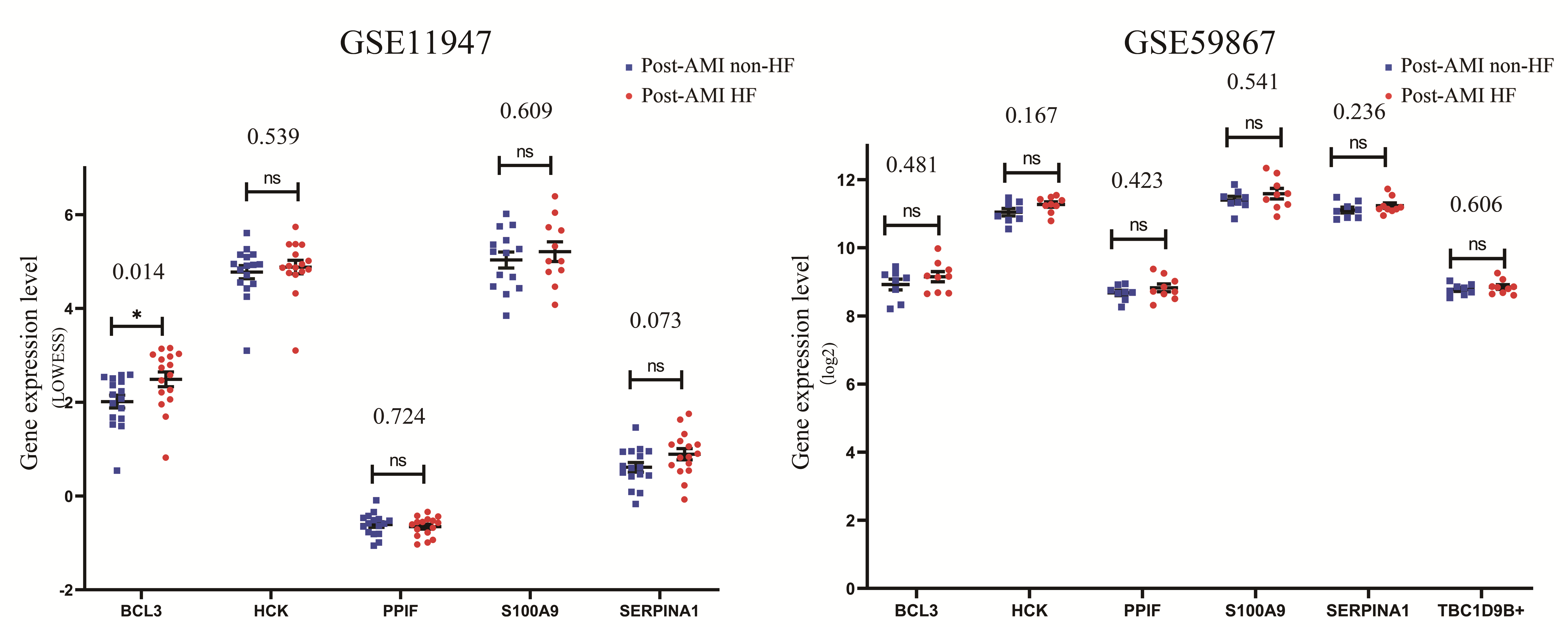

Supplement: Supplementary Figure 6 — Validation of the expression levels of real hubgenes of Niu’s study in post-AMI HF and post-AMI non-HF patients based on datasets GSE11947 and GSE59867. [file Image_6.tif]
